# Size and shape of the neurocranium of laying chicken breeds

**DOI:** 10.1016/j.psj.2024.104008

**Published:** 2024-06-22

**Authors:** Barış Can Güzel, Nicoleta Manuta, Burak Ünal, Iliana Stefanova Ruzhanova-Gospodinova, Sokol Duro, Ozan Gündemir, Tomasz Szara

**Affiliations:** ⁎ Department of Anatomy, Faculty of Veterinary Medicine, Siirt University, Siirt 56100, Türkiye; † Institute of Graduate Studies, Istanbul University-Cerrahpasa, Istanbul 34320, Türkiye; ‡ Department of Anatomy, Faculty of Veterinary Medicine, Istanbul University-Cerrahpasa, Istanbul 34320, Türkiye; § Department of Anatomy, Physiology and Animal Sciences, University of Forestry, Sofia 1797, Bulgaria; # Department of Anatomy, Faculty of Veterinary Medicine, Agricultural University of Tirana, 1029 Tirana, Albania; ‖ Department of Morphological Sciences, Institute of Veterinary Medicine, Warsaw University of Life Sciences, Warsaw, Poland

**Keywords:** avian anatomy, cranium, geometric morphometric

## Abstract

The neurocranium in birds provides valuable insights into their morphological diversity, including adaptations related to brain size, facial shaping, and environmental factors. This study analyzes the neurocranial shape characteristics and size of chickens with similar genetic backgrounds. By examining the neurocranial shape variation in chickens of the same age and sex, the study aims to understand the factors contributing to morphological diversity within this specific group. 3D geometric morphometrics was used to analyze 235 neurocrania from four chicken breeds. The analysis revealed significant differences in centroid size among the chicken breeds. The largest neurocranium centroid size was found in Sasso chickens., which were statistically separated from Atak-S. Additionally, centroid size effectively differentiates between Lohmann Brown and Lohmann Sandy chicken breeds. The most significant shape variation concerned the width of the rostral part of the frontal bone. However, according to the PC1 value, the shape variation was observed within rather than between groups. Lohmann Sandy chickens exhibited higher variability in neurocranial shape, suggesting greater shape diversity within this breed than others. As for shape analysis, the breeds showed closer similarity to each other. Lohmann Sandy chickens are characterized by positive PC1 value, with the rostral end of the frontal region inclined more ventrally, and a more extensive basioccipital region. Sasso chickens have a more dome-shaped middle part of the frontal region than other breeds. The study also identified the most significant shape variation among the study samples, observed at the rostral part of the frontal bone. These findings contribute to understanding the genetic and environmental influences shaping neurocranial morphology in chickens. Similar studies in different bird species and subspecies offer valuable insights into avian biology and adaptation.

## INTRODUCTION

In contrast to mammals, the avian skeleton is uniquely adapted to active flight, a capability crucial for the survival and conservation of bird species. Flight enables birds to access food sources, that are unreachable by terrestrial vertebrates and facilitates their seasonal migrations to optimal feeding grounds (Baumel and Witmer, 1993).

The skeletal structure is important due to its relevance in phylogenetic and taxonomic studies. Moreover, skeletal disorders can result in substantial financial losses for the poultry industry (King and McLelland 1975).

Birds exhibit some of the most specialized skulls among all vertebrates (Feduccia, 1975). Notably, avian skulls are characterized by diverse shapes and variable dimensions (Zusi, 1993). As in other vertebrates, the skull of birds consists of a neurocranium and a splanchnocranium (Nickel et al., 1977; König et al., 2016). The neurocranium includes several bones such as the os occipitale, os sphenoidale, os squamosum, os parietale, os frontale, paired ossa otica, unpaired os mesethmoidale, os ectethmoidale, and os lacrimale (Koch, 1973). The orbits exhibit notable size, being extensively spaced apart by a slender interorbital septum, while the braincase tends to be relatively voluminous, particularly in smaller bird species. A single occipital condyle connects the skull to the vertebral column, enabling a broad range of head movements not observed in other vertebrates. The configuration of the facial skeleton is dictated by the morphology of the beak, with a distinctive ability to elevate the upper portion of the beak owing to its flexible connection with the cranium. This flexibility is facilitated by the maxillopalatine apparatus, with the os quadratum serving as its central component. Functionally analogous to the mammalian temporomandibular joint, this apparatus enables the avian beak's dynamic movement (Nickel et al., 1977; Szara et al., 2022).

Significant theoretical advancements have been made regarding various mathematical spaces crucial for analyzing the statistics of shape change (Goodall, 1991). These advancements explore the impacts of linear transformations applied to the coordinates of landmarks whether on specimens or references on diverse statistical analyses. These studies also investigate how such transformations affect estimates of uniform and nonaffine components of shape variation (Rohlf 1996). Numerous researchers have investigated avian skull morphology, spanning various bird species such penguins (Hospitaleche and Tambussi, 2006; Hospitaleche, 2009; Szara et al., 2023), skua (Hospitaleche et al., 2009), rock pigeon (Szara et al., 2024) yellow-legged gull (Pazvant et al., 2022) and tinamidae (Degrange and Picasso, 2010). Some of these studies have used geometric morphometric analysis methods (Gündemir et al., 2020).

The neurocranium in birds offers valuable insights into their morphological diversity, reflecting brain size, facial bone shaping, and environmental adaptations. Previous studies have indicated that a domed neurocranium is often associated with a retracted upper beak or facial structure, highlighting the intricate relationship between form and function in avian evolution (Hofer, 1952; Marugán-Lobón et al., 2022). According to the Plateau and Foth (2021), the chicken neurocranium fully matures in the first months of their life.

Researchers such as Marugán-Lobón and Buscalioni (2006) have explored the relationship between brain morphology and skull dimensions, including the cranial base and foramen magnum, suggesting that changes in the avian neurocranium are closely tied to variations in overall skull size. Furthermore, investigations into neurocranial morphology by Gündemir (2019), Sridevi et al. (2020), and Tokita et al. (2017) have highlighted significant diversity in structure and shape among different bird species. Stange et al. (2018) conducted a detailed examination of neurocranial morphology in chicken subspecies, emphasizing marked differences and noting that neural crest-derived skull parts exhibit substantial variation in chickens. These studies collectively underscore the complexity of avian neurocranial morphology and its importance in understanding avian evolution and adaptation.

Our study focuses on the neurocranial shape characteristics and size of chickens with similar genetic backgrounds. By examining neurocranial shape variation in chickens of the same age and sex, the study aims to elucidate the underlying factors contributing to morphological diversity in this specific group. It attempts to explain whether the relatedness of laying poultry breeds is reflected in the shape of the skull. This approach provides a unique perspective on the genetic and environmental influences shaping neurocranial morphology in chickens, offering valuable insights into avian biology and adaptation.

## MATERIAL AND METHODS

### Ethical Statement

The research material was obtained from the slaughterhouse. No animals were harmed for this study. Since slaughterhouse materials are not subject to ethics committee permission, an ethics committee decision is not required for this study.

### Samples

This study used 58 Atak-S (**AT**), 54 Sasso (**SS**), 63 Lohmann Brown (**LB**), and 60 Lohmann Sandy (**LS**) chickens, all 5 to 6-mo-old males. Samples with pathological morphological disorders were excluded from the study. After slaughter, the skulls were collected and subjected to maceration to remove the skin and muscle layers. The skulls were then boiled for 30 min and soaked in 35% hydrogen peroxide for 10 min to remove fatty tissue. Following these steps, the facial portions of the skulls were removed, and the resulting neurocraniums were allowed to dry at room temperature in a well-ventilated area for 10 d.

### Modelling

The skulls from the samples underwent 3D modeling using the Shining 3D EinScan SP 3D scanner (Shining 3D Hangzhou, China). Fixed scanning was performed, employing a rotary table, with a dot interval of 0.2 mm. After the scanning procedure, the data obtained was processed with EXScan software (Shining 3D Hangzhou, China) for mesh operations, and the resulting models were saved in PLY format for further analysis.

### Data

The study employed 3D geometric morphometric analysis, which commenced with developing an initial landmark set. This process involved utilizing the PseudoLMGenerator module within the 3D Slicer program (version 5.2.2). Points were meticulously distributed on both sides of the neurocranium using a plane, ensuring even spacing with a tolerance of 9%. A template was then generated based on this distribution, dictating the shape of the estimated sampling template.

**Figure 1:**
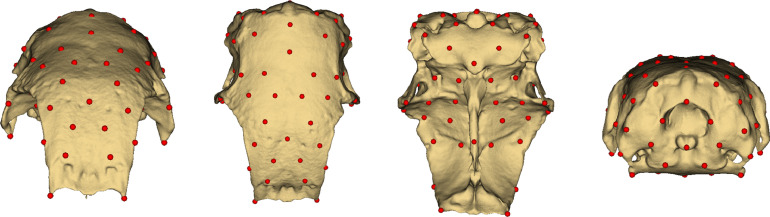
Landmarks.

Following the creation of the template, landmarks were placed on the surface of the neurocranium. Subsequently, the regularity of the sampling was refined to achieve a more uniform distribution of landmarks, culminating in the final landmark set illustrated in Figure 1. The study established a comprehensive set of 89 landmarks through these procedures.

Automated Landmarking through Point Cloud Alignment and Correspondence Analysis (**ALPACA**) was employed in the study to process the initial draft landmark set across all other 3D models (Porto et al. 2021). This module within the Slicer program (version 5.2.2), automatically applies the draft landmark set to all samples. By doing so, ALPACA facilitated a more efficient landmarking process while ensuring consistency across the entire dataset (Rolfe et al. 2021).

ALPACA operates by aligning the point clouds of the 3D models and identifying corresponding points across these models. This automated approach minimizes the potential for human error and enhances the analysis of large datasets. Moreover, ALPACA impacts consistent landmark distribution, crucial for accurate morphometric analysis.

### Statistical Analysis

In this research, Principal Component Analysis (**PCA**) played a pivotal role in uncovering underlying patterns within the data while effectively reducing its complexity without sacrificing significant variability (Boz et al. 2023). Specifically applied to the landmark data derived from neurocranium, PCA allowed for the identification of primary axes (principal components) along which variations in neurocranium shape manifested. By visualizing these components through graphical representation, researchers gained insights into how neurocranium shapes varied across chicken breeds. To elaborate, PCA essentially simplifies the complex relationships among multiple variables into a smaller set of meaningful dimensions called principal components. These components capture the maximum variance in the data, enabling researchers to grasp the main trends or patterns present. In the context of this study, PCA enabled the exploration of how different breeds of chicken exhibited distinct neurocranium shapes, shedding light on potential morphological differences. Furthermore, the positive and negative limits of the principal components were visualized using 3D models of the neurocranium. This visualization technique involved deforming the neurocranium models based on the values of the principal components, providing a tangible representation of how shape variations correspond to changes in the principal component scores.

Moving on to centroid size and Procrustes distance, these metrics provided additional insights into the size and shape characteristics of the neurocranium samples. Centroid size, calculated as the square root of the sum of squared distances of each landmark from the centroid of an object, served as a comprehensive measure of overall size based on the landmark configuration. Meanwhile, Procrustes distance quantified the extent to which each sample deviated from the average shape derived from the analysis. Analysis of Variance (**ANOVA**) was employed in the study to statistically examine the differences in centroid size between different chicken breeds. Given that the number of samples in each breed was not equal, the Bonferroni test was utilized to address this imbalance and ensure the validity of the statistical analysis.

## RESULTS

A summary statistics of the centroid size is provided in Table 1 for each chicken breed, including the number of samples, the range of centroid sizes (minimum and maximum), the total sum of centroid sizes, the mean centroid size, and the standard deviation of centroid sizes. These values give an overview of the centroid size distribution within each breed, highlighting differences in size variability and average size among the breeds.

**Table 1:** The number of samples (N), minimum, maximum, mean, and standard deviation for centroid sizes of different chicken breeds.

Data	Atak-S	Lohmann Brown	Lohmann Sandy	Sasso
**N**	58	63	60	54
**Min**	129.132	134.4006	127.5561	137.5504
**Max**	151.3229	151.0661	147.3762	186.2112
**Mean**	139.1317	140.5133	137.6322	146.59
**Stand. dev**	4.002454	3.157875	4.368984	9.740039

The analysis of centroid size differences revealed several significant distinctions, indicating varying sizes across breeds (Table 2). Significant differences were notably observed between Atak-S and Sasso chickens. (*p* < 0.001) indicating that centroid size effectively differentiates these two breeds. Similarly, a significant difference was found between Lohmann Brown and Lohmann Sandy chickens, with Lohmann Brown chickens showing a larger centroid size than Lohmann Sandy chickens (*p* = 0.031). This suggests that centroid size can also effectively differentiate between Lohmann Brown and Sandy breeds based on size.

**Table 2:** The pairwise comparisons of centroid size differences between different chicken breeds.

Pair	Difference	SE	Q	Lower CI	Upper CI	Critical Mean	p-value
**AT-LB**	1.3817	0.7422	1.8615	-1.3346	4.098	2.7163	0.554
**AT-LS**	1.4994	0.7511	1.9964	-1.2492	4.2481	2.7487	0.493
**AT-SS**	7.4584	0.7713	9.67	4.6356	10.2811	2.8227	p < 0.001
**LB-LS**	2.8811	0.7357	3.9159	0.1884	5.5737	2.6926	p < 0.05
**LB-SS**	6.0767	0.7564	8.0339	3.3085	8.8449	2.7682	p < 0.001
**LS-SS**	8.9578	0.7651	11.7085	6.1578	11.7577	2.7999	p < 0.001

Pair: This column indicates the pair of chicken breeds being compared.

SE: Standard Error, which indicates the precision of the estimated difference.

Q: The Q value, which is a measure of the standardized difference between the two groups.

Lower CI: The lower bound of the 95% confidence interval for the difference in centroid size.

Upper CI: The upper bound of the 95% confidence interval for the difference in centroid size.

Critical Mean: The critical mean for the comparison.

Furthermore, there were significant differences between Lohmann Sandy and Sasso chickens, with Lohmann Sandy chickens having a smaller centroid size than Sasso chickens (*p* < 0.001). This implies that centroid size is a valuable metric for distinguishing between these breeds based on their overall size variations. These findings suggest that centroid size is a reliable indicator of size variations among chicken breeds. This is especially evident when distinguishing between Atak-S and Sasso chickens and between Lohmann Brown and Lohmann Sandy chickens.

**Figure 2:**
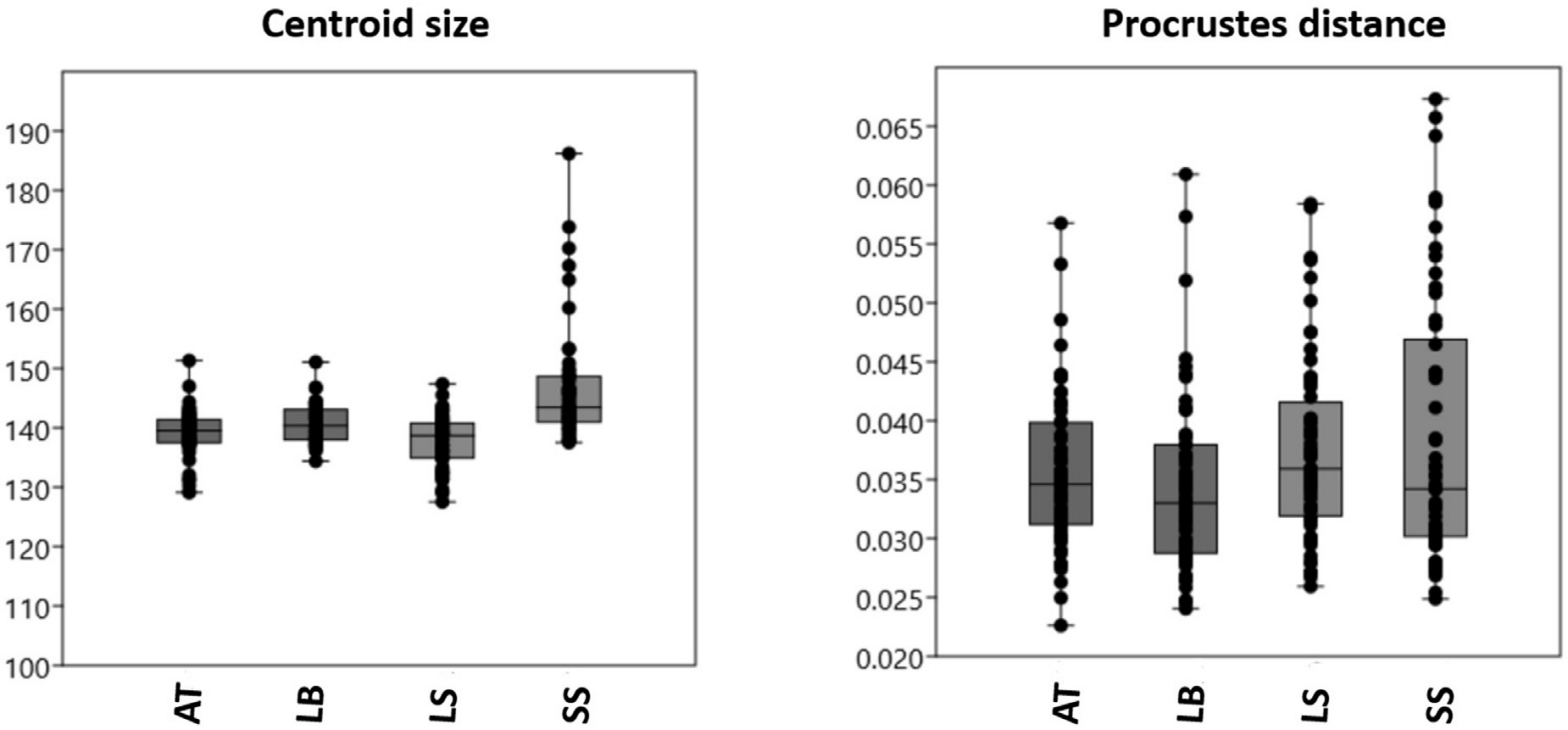
Procrustes distance and centroid size values. The darker horizontal line is the median, the margins of the boxes represent the percentiles (25 and 75), and the extensions of the bars represent maximal and minimal values for chicken breeds.

The graphs of the Procrustes distance and centroid size values for the breeds are displayed in Figure 2. Based on these results, the Lohmann Brown and Sasso chickens showed the highest shape variation, as indicated by their higher Procrustes distance. This suggests that there are more pronounced shape differences between these 2 breeds compared to the other pairs.

**Figure 3:**
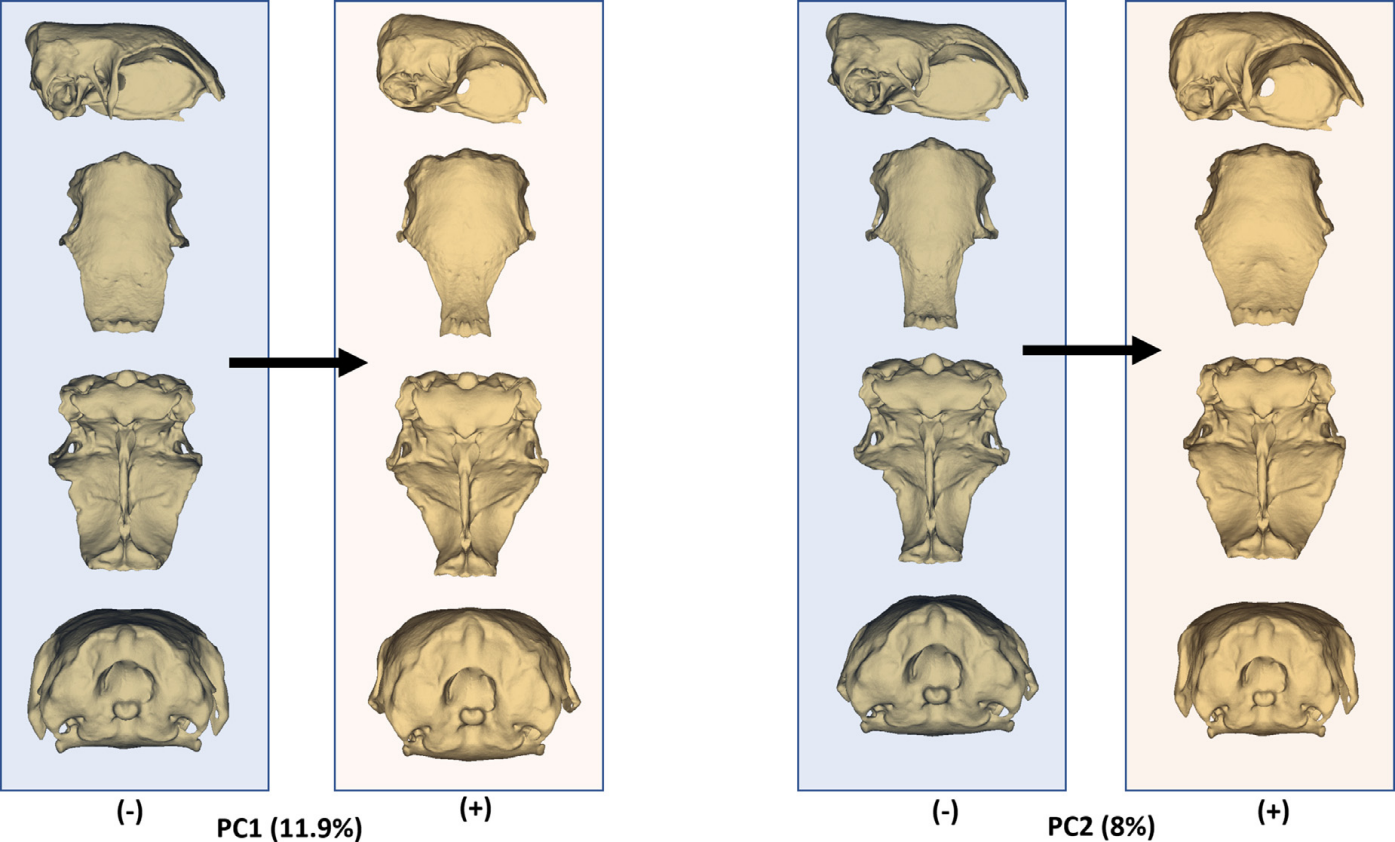
Models describing neurocranium shape between the negative and positive values of PC1 and PC2 from lateral, dorsal, ventral, and caudal views, respectively.

Figure 3 depicts changes in neurocranium shape at negative and positive values of PC1 and PC2. A negative PC1 value corresponded to a wider rostral part of the frontal bone, whereas a positive value indicated a narrower one. However, the caudal part of the frontal region was wider at positive PC1 values. Additionally, at a positive PC1 value, the rostral part of the frontal region inclined more ventrally. The basioccipital region exhibited greater development in shape at positive PC1 values.

In PC2, the most significant changes were observed in the frontal region. A positive PC2 value was associated with a dome-shaped middle part of the frontal region. Furthermore, at a positive PC2 value, the anterior part of the frontal bone and orbital margins appeared wider. Lastly, at a positive PC2 value, the parietal region exhibited a more oval shape.

**Figure 4:**
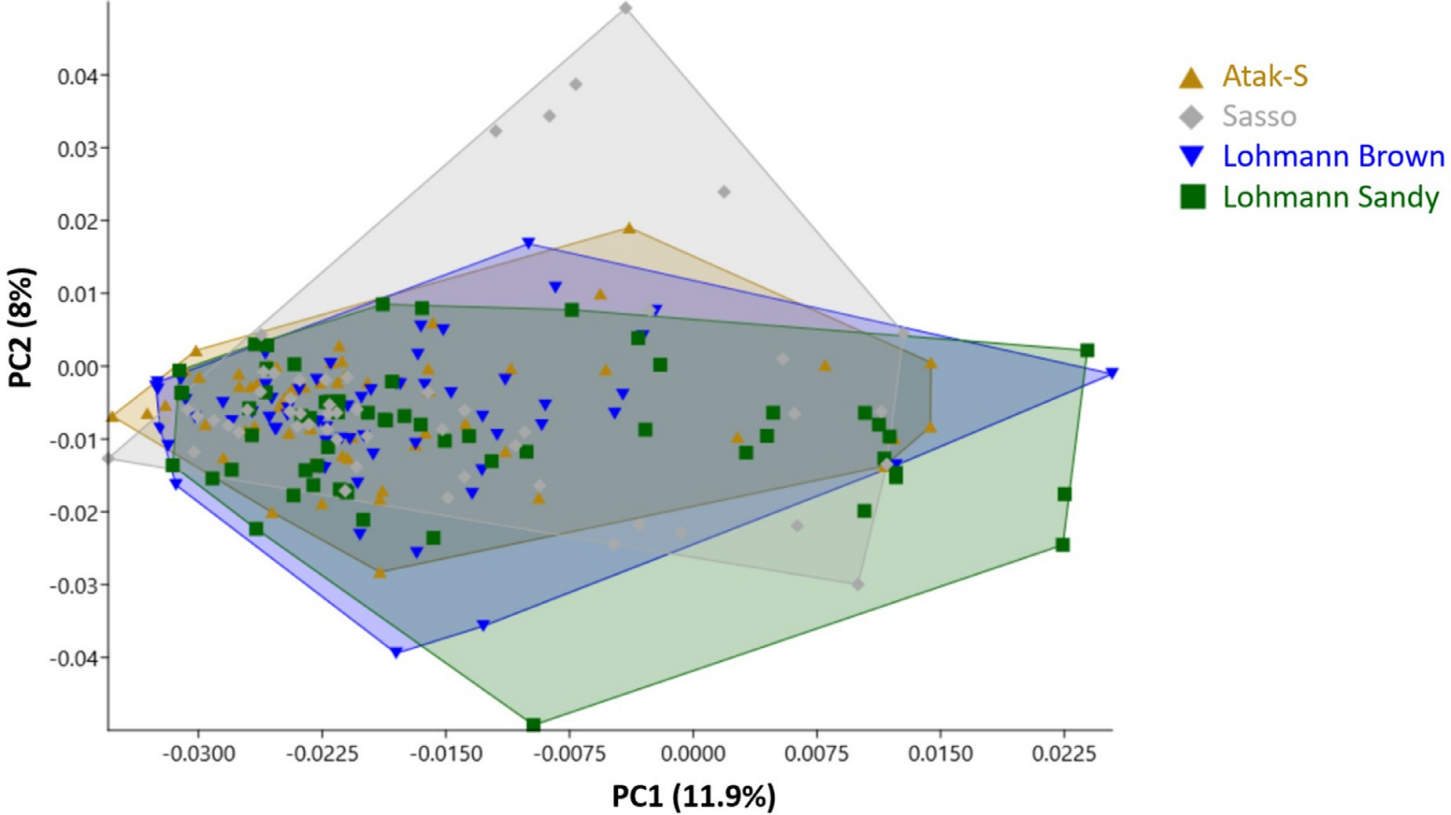
Principal component analysis scatter plot comparing neurocranial morphology of the chicken breeds with convex hulls.

Based on PC1 values, Lohmann Sandy chickens have the highest variation in PC1 values, as indicated by their higher standard deviation of 0.0154. The PC1 values for Lohmann Sandy chickens range from -0.0316 to 0.0239, indicating a similar range of variation as Lohmann Brown chickens. However, the higher standard deviation of 0.0154 suggests more variability in PC1 values among Lohmann Sandy individuals than Lohmann Brown chickens (Figure 4). The PC1 values for Sasso chickens range from -0.0355 to 0.0128 and from -0.0352 to 0.0144 for Atak-S. Based on these values, Sasso chickens have the highest variation in PC2 values, as indicated by their higher standard deviation of 0.0150. This suggests that Sasso chickens exhibit more variability in neurocranium shape along PC2 compared to the other breeds.

In summary, we found differences between breeds of laying hens. These relate to the size rather than the shape of the cranium. Closely related chickens like Lohmann Sandy and Lohmann Brown show more similarities, but the differences between Atak-S and Sasso chickens are also subtle.

## DISCUSSION

The research found that size differences in the neurocranium were more decisive in distinguishing between chicken breeds. Significant differences in centroid size were observed among the various breeds, indicating variability in size. However, in shape analysis, the breeds showed closer variation to each other. This suggests that while there were significant size differences, the shapes of the neurocranium were more similar across breeds. Interestingly, Lohmann Sandy chickens exhibited higher variability in neurocranial shape along PC1, indicating greater shape diversity within this breed relative to others. As a result of the study, the most significant shape variation between the analyzed samples was seen at the anterior end of the frontal bone.

The low variation explained by PC1 (11.9%) and PC2 (8%) in the study results may arise from several factors. The relatively low variation explained by PC1 and PC2, even with a large dataset, implies that numerous factors influence the shape of the neurocranium. These factors likely include genetic, developmental, and environmental determinants, making it challenging to capture them fully with a limited number of principal components. Furthermore, the morphological similarity of the chicken breeds studied could contribute to the lower variation explained. In cases where the breeds are genetically related or exposed to similar environmental influences, the overall shape variation in the neurocranium may become more subtle, resulting in less variation explained by the principal components (Ino et al., 2008).

The skeletal system of chickens, particularly the neurocranium, can exhibit morphological changes in response to their behavioral adaptations to environmental stimuli (Mehlhorn and Caspers, 2021). These adaptations may include adjustments to physical aspects of the environment, such as perching structures or feeding mechanisms, as well as social interactions within the flock. Even though there were individual variations in neurocranial shape, these differences were poorly effective in separating breeds (Usman et al., 2020). However, the analysis did reveal that size was a distinguishing feature among some breeds. While genetic and environmental factors may influence skeletal morphology, other factors such as size may play a more significant role in distinguishing between chicken breeds in similar environments.

The morphometric characteristics of chickens have been reported to be determinative among species in several studies (Ino et al., 2008; Dana et al., 2010). These studies indicate that body weight and other linear morphometric data can vary between sexes (Assefa and Melesse, 2018). The current study yielded significant findings across four distinct laying chicken breeds. Notably, the neurocranium size of Sasso chickens surpassed that of the other breeds, with this variance being statistically significant. As Sasso is a dual-purpose chicken breed, we posit that this trait may exert a notable influence on the overall morphological characteristics of the body, including the neurocranium, particularly when compared to conventional laying hen breeds (Duro et al., 2024). The size difference between Atak-S and Lohmann Brown chickens, as well as between Atak-S and Lohmann Sandy chickens, was nonsignificant.

In general, size or morphometric results are successful in distinguishing between breeds.

Various studies have been conducted to examine different chicken breeds and identify morphological differences in certain parts of the skeleton. Ino et al. (2008) studied 11 chicken breeds and found that the region around the junction of the neurocranium and the viscerocranium played a significant role in morphological differences among breeds, particularly in PC1 and PC2. Verdiglione and Rizzi (2018) reported notable individual skull variations in the frontal bone in Padovana chickens. Similarly, Stange et al. (2018) highlighted the cranial vault, formed by dermal and neural crest-derived bones, as the most variable part of the chicken skull. The results of the present study are consistent with previous research, particularly those of Strange's findings. The PCA analysis conducted on four different chicken breeds revealed that a significant portion of the shape changes in PC1 and PC2 were attributed to the frontal bone. PC1 characterized the narrower rostral edge of the frontal bone, while PC2 explained variation in the cranial vault. These results suggest that specific skull regions, such as the frontal bone and cranial vault, play a crucial role in shaping morphological differences among chicken breeds. The diversity of variation observed in the study may be related to the specific chicken breeds selected. Different chicken breeds may exhibit unique shape variations in the analyses due to their genetic background, developmental history, and environmental influences. Breeding practices, specific trait selection, and adaptation to different environments can all contribute to morphological diversity among breeds. Therefore, conducting similar analyses in different chicken breeds may reveal varying patterns of shape variation, highlighting the importance of considering breed-specific characteristics in morphological studies.

Based on the discussions provided, the study offers valuable insights into the variations in neurocranial size and shape among different chicken breeds. The research indicates that while there are significant differences in centroid size, suggesting variability in size among breeds, the shapes of the neurocranium are more similar across breeds. This proves that size may be more important in distinguishing between chicken breeds in similar environments than shape. Conducting similar analyses in different chicken breeds may reveal varying patterns of shape variation, providing further insights into avian skull anatomy.

The limitation of our work is the lack of female individuals and birds at various stages of development. We also did not have accurate data on body weight or nutritional conditions. It can be expected that these factors influence the morphological features of the head skeleton.

## DISCLOSURES

The authors declare no conflicts of interest.
